# Physicochemical Quality Characteristics of Southeastern Anatolia Honey, Turkey

**DOI:** 10.1155/2020/8810029

**Published:** 2020-09-01

**Authors:** Semra Gürbüz, Neslihan Çakıcı, Serdar Mehmetoğlu, Hilal Atmaca, Tahsin Demir, Mukaddes Arıgül Apan, Ömer Faruk Atmaca, Fazıl Güney

**Affiliations:** ^1^Gastronomy and Culinary Arts, Faculty of Tourism, Mardin Artuklu University, Mardin 47080, Turkey; ^2^Ministry of Agriculture and Forestry, Directorate of Beekeeping Research Institute, Ordu 52850, Turkey

## Abstract

This study was performed to investigate the physicochemical quality characteristics of honey produced in Southeastern Anatolia of Turkey. A total of 68 honey samples collected from different beekeepers were analyzed for sugar components, moisture, pH, HMF, electrical conductivity, free acidity, proline values, and diastase number using the methods recommended by the International Honey Commission. The color value was determined by the Hanna HI 96785 color identification device using the Pfund scale. The mean values of fructose + glucose, fructose/glucose ratio, sucrose, and maltose were 70.97 ± 3.27%, 1.21 ± 0.15, 0.90 ± 1.35%, and 2.88 ± 1.42%, respectively. The moisture, pH, electrical conductivity, free acidity, diastase number, proline, and HMF values were 15.91 ± 1.05%, 4.10 ± 0.73, 0.21 ± 0.04 mS/cm, 14.94 ± 6.81 meq/kg, 10.68 ± 4.61, 420±, 174 mg/kg, and 18.5 ± 31.43 mg/kg, respectively. All of the samples met the international standards and legal limits set in Turkey for fructose + glucose, sucrose, moisture, electrical conductivity, and free acidity, whereas 20.58%, 25%, 10.29%, and 8.82% of the samples did not meet the standards and legal limits for the diastase number, proline value, HMF value, and fructose/glucose ratio, respectively. It has been considered to be important to raise awareness of the producer about good production practices and to ensure continuity of inspections for high-quality honey production.

## 1. Introduction

Honey, which has antibacterial, antioxidant, and anti-inflammatory properties, special taste, and aroma, is highly nutritious natural food [1–3]. The content of honey consists of 70–80% carbohydrates, 10–20% water, and small amounts of enzymes, proteins, hormones, vitamins, amino acids, phenolic compounds, pollen particles, essential oils, and sterols [3, 4]. The composition of honey varies depending on the plant source, bee type, geographical origin, climatic conditions, seasons, harvest, processing, and storage conditions [5]. Honey quality is determined by chemical, physical, microbiological, and sensory characteristics [6]. The limits for honey composition and quality factors were determined by the Codex Alimentarius Commission (CAC) Honey Standard for the quality and safety of honey, as well as fair international honey trade [7]. Regulations are available in the European Union (EU) and in Turkey [8, 9]. In the Turkish Food Codex (TFC) Honey Communiqué, flower honey is defined as honey obtained from plant nectars. Moisture up to 20%, free acidity up to 50 meq/kg, electrical conductivity up to 0.8 mS/cm, hydroxymethylfurfural (HMF) up to 40 mg/kg, sucrose up to 5 g/100 g, diastase number at least 8, proline value at least 300 mg/kg, total fructose and glucose at least 60 g in 100 g honey, and fructose/glucose ratio as 0.9–1.4 were the limits for flower honey [9].

Turkey is among the largest honey-producing countries in the world because of its suitable geographical conditions and climate for beekeeping. Honey production of Turkey was 114,471 tons in 2017 [10]. There is an increasing trend in the Southeastern Anatolia Region to beekeeping, which is an important source of income in the rural areas, where plant flora is rich [11, 12]. Geographical Sign Registration Certificate was received from Turkish Patent Institute in 2003 for Pervari honey, which is produced in this region [13].

This study was aimed to determine the physicochemical quality characteristics such as moisture, pH, free acidity, diastase activity, proline, HMF, electrical conductivity, and color values and fructose, glucose, sucrose, and maltose amount of honey produced in 9 regions located in Southeastern Anatolia of Turkey and to evaluate their compliance with legal regulations and standards.

## 2. Materials and Methods

### 2.1. Honey Samples

In this study, a total of 68 floral honey samples were used. The samples were collected from Faraşin (19), Uludere (5), Idil (3), Besta (3), Silopi (1), Pervari (17), Siirt Center (13), Tillo (5), and Şirvan (2) regions located in Southeastern Anatolia of Turkey. Honey samples were collected directly from beekeepers in 2018. Approximately 350 g of filtered honey samples was taken from each producer in glass cobs and stored at room temperature until analysis.

## 3. Methods

To determine the sugar composition, 5 g of the honey sample was weighed and dissolved in some distilled water. 25 ml of methanol was added, and the flask was made up to 100 mL. The solution was filtered through a 0.45 syringe filter and read on the high-performance liquid chromatography (HPLC) instrument (Thermo Scientific UltiMate 3000; Thermo Scientific Amino Gold column) with the RID detector. Fructose, glucose, sucrose, and maltose (Sigma-Aldrich) standards were used for the calibration curve. The mobile phase was acetonitrile:distilled water (80:20, v/v) with 1.3 mL/min flow rate. Column temperature was 30°C. The moisture value was determined by a refractometer (Atago RX5000*α*). pH of honey was determined by a pH meter (Hanna/HI2030-02). Electrical conductivity of honey was measured by a conductimeter (Hanna/HI2030-02). Free acidity was determined by the titrimetric method. To determine the HMF value, 10 g of the honey sample was weighed and dissolved in some distilled water. 25 ml of methanol was added, and the flask was made up to 50 mL. The solution was filtered through a 0.45 syringe filter and read on the HPLC instrument (with Thermo Scientific UltiMate 3000; Thermo Scientific Amino Gold column) with the DAD detector. Fructose, glucose, sucrose, and maltose (Dr. Ehrenstorfer) standards were used for the calibration curve. The mobile phase was methanol:distillated water (10:90, v/v) with 1.0 mL/min flow rate. Column temperature was 30°C. Diastase activity and proline analysis were determined by a spectrophotometer (PerkinElmer Lambda 25). All the analyses mentioned above were realized according to the methods recommended by the International Honey Commission (IHC) [14]. Color was determined using the Pfund scale with the Hanna HI 96785 color determination device [15]. All analyses were performed duplicate, and mean of the duplicate data was used in statistical analyses. Silopi was not included in the statistical analysis due to the number of samples.

### 3.1. Statistical Analysis

Statistical analysis was performed by SPSS ver. 21 package program. The differences between the sampling regions for the physicochemical values obtained from the analyzed samples were determined by one-way ANOVA, Tukey, and Games–Howell tests. In the analyses, *P* < 0.05 was considered statistically significant.

## 4. Results and Discussion

**Figure 1:**
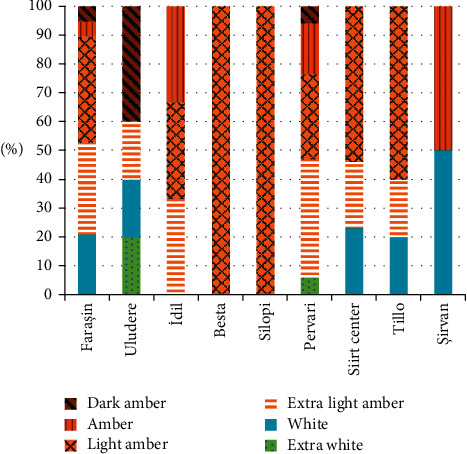
Color distribution of honey according to sampling locations.

The mean, standard deviation, and minimum and maximum values of fructose, glucose, fructose + glucose, fructose/glucose, sucrose, and maltose in honey samples according to sampling regions are shown in Table 1. The moisture, pH, electrical conductivity, number of diastase, HMF, and proline values are given in Table 2, and the results of color analysis are given in Figure 1.

Fructose and glucose are the main sugars in honey. Although the composition of the sugars in honey depends on the plant flora, it is also affected by the geographical conditions and climate [5, 16, 17]. In this study, the mean fructose + glucose value of honey samples was determined as 70.97 ± 3.27%, which was ranging from 62.55 to 77.25%. This value was higher than 60/100 g, which is indicated as the minimum value by the CAC Honey Standard, TFC Honey Communiqué, and EU directive of 2001/110/EC [7–9]. Our result was lower than the result of Vit et al. [18] and close to the result reported by Kahraman et al. [19], whereas it was partly higher than that reported by some other researchers [6, 20, 21]. Statistically significant differences for the glucose ratio were found in some sampling locations. The glucose value in the samples taken from Faraşin was higher than the samples collected from Siirt Center and Tillo. The glucose value in the samples taken from İdil was higher than Siirt Center and Tillo (Table 1). Differences between locations could possibly be due to nectar content and environmental conditions.

It has been stated that the ratio of fructose/glucose, which is used in the evaluation of the crystallization degree of honey, is related to nectar used in the production of honey [4, 16]. In our study, the mean fructose/glucose ratio was 1.21 ± 0.15 (1.03–1.67). Six (8.82%) of the analyzed samples were found to be above the legally determined maximum value of 1.4. Improper values were found in honey samples collected from Pervari (2), Siirt Center (3), and Tillo (1). Concerning the ratio of fructose/glucose values, it was found that there was a statistically significant difference between Faraşin and Siirt Center sampling locations (Table 1). The ratio of the fructose/glucose value in Siirt Center was high.

Sucrose and maltose are sugars found in small amounts in honey. High sucrose in honey is seen when honey is harvested before sugar is completely transformed into glucose and fructose by the effect of the invertase enzyme [1, 22] or is seen in the case of overfeeding of bees with sugar in spring [16, 23]. In this study, sucrose was not detected in 38 (55.88%) of the samples. The sucrose contents of the other samples were less than the legally permissible maximum value of 5%. The mean value of 0.90 ± 1.35% (nd-4.10%) for the sucrose content, which was found in the present study, was lower than the sucrose content found by some other researchers [6, 24]. However, the mean sucrose content of honey in our study was close to the results of Can et al. [17] and Küçük et al. [1].

Statistically significant differences for the maltose value were found in some sampling locations, and the differences are indicated in Table 1. The mean maltose value of 2.88 ± 1.42% that was determined in this study was higher than the maltose values of 1.05 ± 0.87% and 0.31 ± 0.02% reported by Can et al. [17] and Habib et al. [25], respectively. On the contrary, our result was lower than the finding of Manzanares et al. [26] who found maltose value as 5.31 ± 0.97%.

The moisture content, which is the water amount of honey, is a component of honey that affects viscosity and crystallization properties, as well as honey's flavor, color, and shelf life [5]. Moisture in honey can vary according to the moisture content of the plant used in the production of honey, the harvest season, and climatic conditions [17, 27, 28]. The high water content of honey increases the possibility of fermentation as a result of osmophilic yeast development [2]. Large number of dead yeasts, glycerol, ethanol, and butanediol due to fermentation of honey result in deterioration of its taste [29]. Shelf life of honey with low moisture content is longer. A value above the values determined for moisture in honey on the market is rare. It is reported that water content of less than 20 g/100 g was determined in 91–95% of 30,000 honey samples analyzed between 1989 and 97 [30]. Similarly, all samples analyzed in our study were found to comply with the legal maximum limit of 20% moisture content. The mean moisture content of 15.91 ± 1.05% (ranging from 14.04 to 18.02%) that was determined in our study was consistent with the results of the previous studies [17, 31, 32]. In a study by Erez et al. [33], the moisture value of honey samples taken from three different locations in the Pervari region was determined as 12.58–15.99%. However, in our study, the mean moisture value of honey collected from 17 different producers in the same region was higher than the value found by Erez et al. [33]. This difference may be due to the number of samples and climatic conditions.

The electrical conductivity is related to the concentration of organic acids, proteins, and mineral salts in honey [25]. Since the electrical conductivity of honey depends on the flower, in which the bee receives nectar, it is an important parameter for the determination of flower honey obtained from different flowers [4, 21, 30]. Honeydew honey has higher electrical conductivity than flower honey [17]. In this study, determination of the mean value of 0.21 ± 0.04 mS/cm was found to be compatible with the results of some other researchers [6, 17, 20, 21]. In this study, the value of 0.16–0.03 mS/cm in the Pervari sampling location was consistent with the value detected in honey taken from the Pervari location by Erez et al. [33]. The electrical conductivity values determined in this study were below the legally permitted maximum value of 0.8 mS/cm.

Although there is no legal limit for the pH value of honey, it is desirable to have low pH of honey to avoid microbial contamination [25]. The pH value that varies depending on factors such as processing and storage conditions can affect honey's structure, durability, and shelf life. Because honeydew honey shows higher pH and electrical conductivity compared to flower honey, the pH and electrical conductivity values of honey help to differentiate honeydew and flower honey from each other [34]. Addition of sugar syrup to honey significantly increases pH of honey; thus, pH of honey is an indicator of fraud [4]. In this study, the pH value of analyzed honey samples was found to be acidic (mean 4.10 ± 0.73, ranging from 3.67 to 6.45). Our finding was consistent with the values determined by some other researchers [6, 21, 25].

Free acidity depends on the flower type used by the honey bee for nectar [27]. In the present study, free acidity was found between 14.94 ± 6.81 meq/kg (2.00–44.00). None of the samples exceeded 50 meq/kg, the legal maximum permissible value. In previous studies, Derebaşı et al. [6] and Estevinho et al. [20] reported free acidity values as 24.97 ± 027 and 40.3 meq/kg, respectively, which were higher than the value determined in this study. This difference may be due to the type of flower that the bee receives nectar. In the study by Erez et al. [33], who analyzed honey collected from Pervari, the free acidity value (16.41–26.20 meq/kg) determined in the samples was partly different from the value of 2.00–25.50 meq/kg that was determined in our study in the same location. The difference may be due to the larger sample size in our study.

Hydroxymethylfurfural, which occurs as a decomposition product of monosaccharides found in honey, is not found in fresh honey. It is an important indicator in determining the freshness of honey due to its increased concentration during storage [28, 30]. The HMF value is the main indicator of fraud practices associated with high heating [23]. High diastase activity but low HMF content are expected in high-quality honey. In this study, the mean HMF value was found as 18.50 ± 31.43 mg/kg (1.10–166.25), and this value was lower than the values determined by Vit et al. [18], who found 162.71 ± 184.94 mg/kg HMF values. On the contrary, our finding was consistent with the value of 19.2 ± 2.0 mg/kg determined by Küçük et al. [1] and was higher than the 8.86 ± 0.38 mg/kg value that was determined by Derebaşı et al. [6]. In this study, it was determined that 7 (10.29%) honey samples had higher HMF content than the legally permitted maximum level which is 40 mg/kg. However, the diastase number in the 3 honey samples with high HMF values was below 8, and no negative correlation was found in the others.

Diastase, a heat-sensitive enzyme found in honey, is a parameter that shows freshness of honey and inappropriate heat treatment and storage conditions [4, 16]. A low level of diastase enzyme in honey is an indication that honeybees are fed with glucose [4]. In this study, diastase activity was determined as 10.68 ± 4.61 (0.00–20.60). The value of 17.9 ± 1.3 that was detected in multifloral honey by Küçük et al. [1] was higher than the value we found. The diastase numbers in 14 (20.58%) samples were found to be less than 8, which was determined as the legal limit. The diastase number detected in this study was consistent with the result of Derebaşı et al. [6] but lower than the value of 21.46 determined by Kıvrak et al. [21] in multifloral honey. Detection of the value less than the legal limits may be related to improper heat treatment, storage conditions, and fraud practices with industrial sugar. In this study, the lowest diastase activity value was found in Faraşin and the highest in honey taken from Şirvan. The difference between the results may be due to the number of samples, unsuitable heat treatment, and storage conditions, as well as fraud.

Proline is the most abundant free amino acid in honey due to salivary secretions of honeybees in the process of converting nectar into honey [5]. The proline content of honey is an indicator for determining the quality and antioxidant activity of honey, and it is also used to determine the source of the plant origin of honey [34]. Proline content shows honey ripening status and sometimes sugar fraud applications [30, 35]. Since proline is reduced in fraudulent honey, it is an indicator of honey purity. The amount of proline, which may vary depending on the flora of honey, is also closely related to the working performance of the bee [35]. In this study, the mean of the proline value determined as 420 ± 174 mg/kg (117.15–933.49) was lower than the value 482 ± 160 found by Can et al. [17] in multifloral honey. In this study, proline amount was found less than 300 mg/kg, which was the legal minimum limit, in 17 (25%) of the investigated honey samples. The lowest proline value was determined in Pervari, whereas the highest proline value was found in Tillo.

In this study, no statistically significant difference was found between locations in terms of moisture, pH, electrical conductivity, free acidity, diastase, proline, and HMF values of the analyzed samples (Table 2).

Color of the honey samples varied between 17 and 137 mm Pfund. According to the Pfund scale, color of 2, 10, 19, 27, 6, and 4 of honey samples was extra white, white, extra light amber, light amber, amber, and dark amber, respectively [15]. In Figure 1, the color distribution of honey is given according to sampling locations. Color of honey depends on factors such as botanical origins of honey, heat treatments, mineral content, and contamination with heavy metals, used wax, and duration of storage [5].

## 5. Conclusions

In this study, the physicochemical properties of honey have been determined in Southeastern Anatolia of Turkey, where beekeeping activities have shown significant improvement in recent years. According to the results obtained in this study, fructose + glucose and sucrose contents, moisture, electrical conductivity, and free acidity of the analyzed samples were in compliance with international standards and the legal limits in Turkey. However, some of the samples did not meet the standards and legal limits for diastase number, proline, and HMF values and the fructose/glucose ratio. The production and marketing of honey in compliance with all standard values are important for the prevention of unfair competition and consumer rights, as well as public health. Therefore, it is important to monitor the quality of honey and to make attempts to raise awareness of the beekeepers about quality production.

## Figures and Tables

**Table 1:** Results of sugar contents of the honey samples according to sampling locations, mean ± std (min-max), and sugar values (%).

Locations (*n*)	Fructose	Glucose	Fructose + glucose	Fructose/glucose	Sucrose	Maltose
Faraşin (19)	37.76 ± 1.54 (35.05–40.65)	34.05 ± 2.35^ab^ (29.40–37.35)	71.82 ± 3.19 (65.60–75.95)	1.11 ± 0.08^e^ (1.03–1.28)	1.59 ± 1.72 (nd-4.10)	3.51 ± 1.69^f^(nd-5.50)
Uludere (5)	38.52 ± 2.20 (36.05–41.35)	33.31 ± 2.07 (30.40–35.50)	71.83 ± 2.73 (68.05–75.35)	1.16 ± 0.11 (1.07–1.36)	1.79 ± 2.04 (nd-4.10)	4.34 ± 0.47^ghij^ (3.65–4.75)
İdil (3)	38.77 ± 1.02 (37.60–39.50)	35.17 ± 2.20^cd^ (32.80–37.15)	73.93 ± 3.13 (70.40–76.35)	1.11 ± 0.05 (1.06–1.15)	0.73 ± 1.27 (nd-2.20)	4.18 ± 0.34^klmn^(3.80–4.45)
Besta (3)	40.05 ± 2.18 (37.65–41.90)	33.30 ± 3.48 (29.70–36.65)	73.35 ± 3.38 (71.20–77.25)	1.21 ± 0.17 (1.11–1.41)	0.73 ± 1.27 (nd-2.20)	3.60 ± 0.83 (2.65–4.15)
Silopi (1)	40.15 ± 0.07 (40.10–40.20)	32.75 ± 0.78 (32.20–33.30)	72.90 ± 3.71 (72.40-73-40)	1.23 ± 0.03 (1.20–1.24)	nd nd	4.10 ± 0.00 (4.10–4.10)
Pervari (17)	39.39 ± 2.79 (34.35–45.51)	31.92 ± 2.00 (27.55–35.70)	71.31 ± 3.04 (63.33–76.21)	1.24 ± 0.14 (1.12–1.65)	0.52 ± 1.00 (nd-3.20)	2.48 ± 1.28^gk^ (0.80–4.80)
Siirt Center (13)	38.89 ± 3.36 (33.89–46.47)	30.39 ± 2.04^ac^ (27.86–34.41)	69.28 ± 2.93 (62.55–74.32)	1.29 ± 0.18 ^e^ (1.06–1.67)	0.39 ± 0.59 (nd-1.89)	1.78 ± 0.66^fhl^ (0.70–2.84)
Tillo (5)	38.39 ± 3.20 (34.43–43.37)	29.22 ± 2.12^bd^ (26.78–31.43)	67.61 ± 2.99 (63.06–70.15)	1.32 ± 0.18 (1.20–1.62)	0.70 ± 0.90 (nd-2.20)	2.12 ± 0.50^im^ (1.38–2.70)
Şirvan (2)	37.87 ± 0.31 (37.65–38.09)	30.52 ± 1.02 (29.80–31.24)	68.39 ± 1.33 (67.45–69.33)	1.24 ± 0.03 (1.22–1.26)	nd nd	2.21 ± 0.15^jn^ (2.10–2.32)

Total, *n* (68)	38.67 ± 2.47 (33.89–46.47)	32.30 ± 2.67 (26.78–37.35)	70.97 ± 3.27 (62.55–77.25)	1.21 ± 0.15 (1.03–1.67)	0.90 ± 1.35 (nd-4.10)	2.88 ± 1.42 (nd-5.50)

*P* value	0.645	0.000	0.020	0.009	0.110	0.000

^*∗*^nd: not detected. The values with the same superscript in the same column differ significantly. *P* values: (a) Faraşin-Siirt Center (0.000), (b) Faraşin-Tillo (0.001), (c) İdil-Siirt Center (0.025), (d) İdil-Tillo (0.010), (e) Faraşin-Siirt Center (0.014), (f): Faraşin-Siirt Center (0.009), (g) Uludere-Pervari (0.002), (h) Uludere-Siirt Center (0.000), (i) Uludere-Tillo (0.001), (j) Uludere-Şirvan (0.003), (k) İdil-Pervari (0.007), (l) İdil-Siirt Center (0.001), (m) İdil-Tillo (0.006), and (n) İdil-Şirvan (0.022).

**Table 2:** Results of physicochemical analysis of honey according to the sampling locations and mean ± std (min-max).

Locations (*n*)	Moisture (%)	pH	Electrical conductivity (mS/cm)	Free acidity (meq/kg)	Diastase activity (⁰ Gothe)	Proline (mg/kg)	HMF (mg/kg)
Faraşin (19)	15.63 ± 1.03 (14.32–18.02)	3.88 ± 0.11 (3.69–4.11)	0.19 ± 0.02 (0.14–0.231)	16.29 ± 7.79 (9.50–44.00)	8.13 ± 3.68 (0.00–13.15)	367.79 ± 88.23 (198.36–496.28)	23.68 ± 44.16 (3.80–166.25)
Uludere (5)	15.12 ± 0.81 (14.58–16.48)	3.97 ± 0.05 (3.91–4.04)	0.20 ± 0.05 (0.14–0.26)	15.00 ± 3.39 (11.50–20.50)	11.32 ± 6.14 (5.60–19.45)	381.67 ± 177.68 (193.12–645.24)	4.56 ± 2.39 (1.30–7.30)
İdil (3)	15.84 ± 0.33 (15.59–16.22)	3.84 ± 0.05 (3.81–3.90)	0.20 ± 0.06 (0.16–0.27)	16.17 ± 5.69 (11.50–22.50)	9.08 ± 5.85 (3.10–14.80)	444.41 ± 190.43 (303.76–661.12)	61.27 ± 80.02 (9.70–153.45)
Besta (3)	16.10 ± 1.09 (15.16–17.30)	3.82 ± 0.10 (3.73–3.92)	0.21 ± 0.02 (0.18–0.24)	18.67 ± 8.28 (10.00–26.50)	11.20 ± 0.71 (10.45–11.85)	447.91 ± 118.20 (321.62–555.90)	34.35 ± 31.03 (9.00–68.95)
Silopi (1)	16.68 ± 0.14 (16.58–16.78)	3.84 ± 0.00 (3.84–3.84)	0.22 ± 0.07 (0.22–0.23)	18.50 ± 0.71 (18.00–19.00)	10.75 ± 0.07 (10.70–10.80)	528.73 ± 3.45 (526.29–531.17)	22.15 ± 0.07 (22.10–22.20)
Pervari (17)	16.11 ± 1.05 (14.04–17.80)	4.09 ± 0.86 (3.70–6.45)	0.21 ± 0.041 (0.16–0.03)	13.82 ± 6.47 (2.00–25.50)	12.26 ± 4.75 (3.60–20.55)	456.98 ± 208.86 (117.15–933.49)	12.88 ± 15.71 (1.10–54.40)
Siirt Center (13)	16.31 ± 1.16 (14.36–17.76)	4.49 ± 1.08 (3.67–6.34)	0.21 ± 0.05 (0.25–0.29)	12.12 ± 6.09 (2.50–21.50)	11.66 ± 4.58 (4.30–20.40)	363.25 ± 172.70 (136.88–736.18)	9.14 ± 7.50 (1.20–24.60)
Tillo (5)	15.86 ± 1.41 (14.20–17.88)	4.37 ± 1.11 (3.72–6.33)	0.23 ± 0.06 (0.18–0.30)	15.50 ± 9.85 (2.50–27.50)	10.88 ± 3.77 (5.70–14.80)	617.61 ± 231.78 (403.24–918.69)	21.89 ± 23.59 (1.40–61.15)
Şirvan (2)	15.91 ± 0.07 (15.87–15.96)	4.22 ± 0.71 (3.71–4.72)	0.25 ± 0.02 (0.23–0.26)	19.25 ± 3.18 (17.00–21.50)	14.65 ± 8.41 (8.70–20.60)	449.55 ± 241.39 (278.86–620.24)	15.08 ± 0.46 (14.75–15.40)

Total (68)	15.91 ± 1.05 (14.04–18.02)	4.10 ± 0.73 (3.67–6.45)	0.21 ± 0.04 (0.15–0.31)	14.94 ± 6.81 (2.00–44.00)	10.68 ± 4.61 (0.00–20.60)	420 ± 174 (117.15–933.49)	18.50 ± 31.43 (1.10–166.25)

*P* value	0.522	0.510	0.701	0.720	0.238	0.189	0.295

## Data Availability

All the data used to support the findings of this study are available from the corresponding author upon request (semragurbuz@artuklu.edu.tr).
